# Instituting interaction: normative transformations in human communicative practices

**DOI:** 10.3389/fpsyg.2014.01057

**Published:** 2014-09-23

**Authors:** John Z. Elias, Kristian Tylén

**Affiliations:** ^1^Department of Philosophy, School of Humanities, University of HertfordshireHatfield, UK; ^2^Department of Aesthetics and Communication, Center for Semiotics, Aarhus UniversityAarhus, Denmark; ^3^Interacting Minds Centre, Aarhus UniversityAarhus, Denmark

**Keywords:** experimental semiotics, normativity, conventionalization, communicative practice, institutionalization

## Abstract

Recent experiments in semiotics and linguistics demonstrate that groups tend to converge on a common set of signs or terms in response to presented problems, experiments which potentially bear on the emergence and establishment of institutional interactions. Taken together, these studies indicate a spectrum, ranging from the spontaneous convergence of communicative practices to their eventual conventionalization, a process which might be described as an implicit *institutionalization* of those practices. However, the emergence of such convergence and conventionalization does not in itself constitute an *institution*, in the strict sense of a social organization partly created and governed by explicit rules. A further step toward institutions proper may occur when others are *instructed* about a task. That is, given task situations which select for successful practices, instructions about such situations make explicit what was tacit practice, instructions which can then be followed *correctly* or *incorrectly*. This transition gives rise to the normative distinction between conditions of *success* versus conditions of *correctness*, a distinction which will be explored and complicated in the course of this paper. Using these experiments as a basis, then, the emergence of institutions will be characterized in evolutionary and normative terms, beginning with our adaptive responses to the selective pressures of certain situational environments, and continuing with our capacity to then shape, constrain, and *institute* those environments to further refine and streamline our problem-solving activity.

## INTRODUCTION

Institutions, understood as societal structures constituted and governed, at least in part, by explicit rules, presuppose a language in which such rules can be formulated and expressed (Searle, 2005, 2010). This point alone indicates an intimate interrelation between our institutional and linguistic activities. Yet this dependency on language might tempt us to picture institutions as somehow magically created by declarative speech acts, conjured, as it were, through the incantations of performative utterances. Such a picture obscures the fact that, prior to the formal declaration of an institution’s existence and the explicit articulation of its structures and functions, various practices, customs, conventions, traditions, etc, comprise the relevant activity that undergoes institutionalization. This development is not a matter of mere historical accrual, but a dynamic process necessary for the evolution of viable institutions. Understanding the emergence of institutions from tacit and fluid practices and processes entails disentangling the interplay between the informal and formal, the implicit and explicit, an interplay centrally involving the use of language in different roles and forms.

Recent studies in semiotics and linguistics offer pertinent insights into the coordinative and organizing power of language (e.g., Garrod et al., 2007; Mills, 2013). A broadly evolutionary framework guides much of this work, with semiotic and linguistic communication conceived as adapting to environmental conditions. These experiments demonstrate that interacting participants, jointly solving a problem, often in the guise of a game, are acutely sensitive to the selective pressures of the situation at hand, converging on common communicative practices and vocabularies without explicit deliberation or decision concerning these practices (Garrod and Doherty, 1994; Fay et al., 2008; Mills, 2011). Participants produce manifold communicative forms in response to the demands of the task, with the task in turn exerting selective pressure on those forms, leading, if successful, to the survival of those most functionally suited to the problem situation. Thus a particular situational environment, defined by a particular problem or set of problems, calls forth and selects for communicative practices fit for that situation. This basic dynamic of fecund generation of communicative forms and their functional selection may be viewed as an engine of specialization, spurring and honing the specialized vocabularies characteristic of specific disciplines.

These themes will be expanded in what follows. We will begin with a review of relevant experimental work on the evolution of communicative systems and signs, with special focus on the *optimally interacting minds* experiments (Bahrami et al., 2010; Fusaroli et al., 2012), which provide an especially promising experimental paradigm to explore the role of language in the formation of institutions. Setting the frame of this field of research, we treat these experiments as a kind of laboratory for larger considerations concerning communication and coordinative activity. Specifically, we claim that linguistic interaction within these situations is more continuous with technique and action than propositional representation. This in turn will entail clarifying the notion of *situation* as it operates in these experimental settings, which, as a corollary, will involve critical pressure placed on the idea of *situation models*, and whether the ecological concept of *affordances* might better explicate the dynamics of joint action within the constraints of situations (Knoblich and Sebanz, 2008).

The roles of *convention* and *instruction* in processes of institutionalization will then be taken up, as fulcrums enabling the transition from conditions of *success* to conditions of *correctness*, thereby tracing the emergence of institutions in terms of the transformation of our *normative* engagements. We start with the poles of spontaneous coordination and explicit instruction, which provide a stark way of sketching these normative distinctions. Between these poles, however, lies a continuum involving *convention* and *conventionalization* of communicative practices. Indeed, the implicit conventionalization of technique, of ways of going about and accomplishing tasks, points to the establishment of standards of correctness independent of explicit declaration and decree (Mills, 2011). A spectrum is thus charted, stretching from the convergence of communicative practices, driven and determined by conditions of success, to the development of convention, involving emerging norms of implicit correctness, to the articulation of instructions, which, for the purposes of this paper, defines a kind of endpoint of explicitly stated standards of correctness. These normative considerations, we emphasize throughout, are inextricably bound up with differences in linguistic interaction.

Undergirding the discussion, running through it as a theme, is a *functionalist* conception of language as acutely adaptive communicative activity (Tylén et al., 2010). More generally, this experimental work exemplifies the dynamics of natural languages as living, evolving systems, teeming in their multifaceted applications, their various uses and forms, with certain terms and turns of phrase in turn selected for use in specific situations, leading to the development of adaptive vocabularies fit for particular purposes, and, eventually, to the specialized discourses of distinct disciplines and institutions. What arises, then, is a view of communicative activity as environmentally and normatively sensitive, with selective pressures comprising situations within which communication may be taken as functional or successful. And with the gradual development of convention, and the eventual introduction of instruction, situations become structured according to standards of *correctness.* Institutionalization, then, is defined by the normative move from *selection* to *sanction* of actions within increasingly intentionally informed environments. This approach to institutions is consonant with recent turns in the cognitive sciences (Clark, 2006; Rowlands, 2010; Hutto and Myin, 2012) in which cognitive capacities are conceived as fundamentally environment-involving, as copings and engagements within the constraints of various environments; as such, this paper is an attempt to apply these concepts to the processes and dynamics of institutions (Gallagher and Crisafi, 2009; Gallagher, 2013). From this perspective, much of our large scale social cognitive activity may be viewed as the deliberate shaping of situations and environments, with the aim of guiding and cultivating the activities occurring within them. In shaping and constraining our environments, we shape and constrain our activities and ourselves.

## SETTING THE SCENE: EXAMPLES AND ELUCIDATIONS FROM EXPERIMENTAL SEMIOTICS

Recent studies in experimental semiotics have investigated the evolutionary aspects of semiotic and linguistic communication (Galantucci, 2009; Galantucci and Garrod, 2010). However, empirical investigation of the evolution of natural languages is inherently problematic, as their evolutionary origins are either difficult to ascertain or completely inaccessible, and certainly not available for experimental manipulation. One way experimental semioticians circumvent this problem is by having participants communicate in graphical media without recourse to conventional linguistic symbols (Galantucci, 2005; Healey et al., 2007; Dale et al., 2011), for instance in scenarios similar to the game Pictionary (Garrod et al., 2007; Fay et al., 2008, 2010). These constraints compel participants to create symbols from scratch, thereby setting up conditions in which the evolution of sign and symbol systems can be observed and analyzed.

In a representative experiment, Garrod et al. (2007) had subjects play a game in which they constructed graphical signs for a pre-established set of items; the game proceeded through several rounds in which players play in pairs, in alternating roles of drawer and identifier. In conditions allowing for interactional feedback, participants produced articulated signs based mainly on iconic resemblance to the referred items. However, through an evolutionary process the signs tended to become simplified and streamlined, reflecting a reduction in their iconic or pictorial character. For instance, in a case from a similar experiment (Fay et al., 2008), the graphical representation for “parliament,” which began as a drawing of a chamber with circular benches and stick figures facing one another, ended as an abstraction of two partial curves with a single small circle in between (Fay et al., 2008, p. 3554). While a residuum of iconicity remained, the representation was no longer identifiable by its iconic resemblance to its referent, and would strike a naive newcomer as completely arbitrary. What appears to have happened is that the reference of each use of the sign became its prior use or tokening: the gradually streamlined sign no longer referred to the concept “parliament” directly through resemblance, but rather to previous episodes of successful communication in the history of the sign’s use; i.e., the abstracted partial curves referred to, reminded recipients of, the more complex representations that occurred before. In other words, a stepwise process occurred of incremental simplification through repeated use, with each increasingly reduced instance linked to its predecessor, resulting in the distillation of an optimally efficient form.

Congruent observations have been made concerning natural languages (Millikan, 2005), supporting the relevance of these experiments to the workings of language at large. And while iconicity in verbal language may not be as obviously evident, recent studies have made the case for its prevalence (Perniss et al., 2010), suggesting a similar tradeoff between complexly iconic and more simplified forms dependent on tacit social coordination and negotiation. Again this speaks to the living and evolving nature of languages, undergoing change as they unfold and adapt in space and time. Indeed these experiments offer something of an artificial window onto possible mechanisms underlying the origins of language, the conditions under which words are forged and formed, and in which they must *succeed* if they are to *survive*. Furthermore, as inherently historical phenomena, words do not simply “pick out” their referents in abstract and static one-to-one referential relations, but rather mean what they do through a temporal process of reliable reproduction and use, grounded in the common knowledge that others in the community are participants in that history. In the experiment above, for example, the simplification of signifiers ensues precisely because participants trust that interlocutors will have encountered something sufficiently like the sign in the past, such that they will recognize the shorthand version on offer. Of course community members do not need to be familiar with all the historical details of a sign’s use: what matters is that those in currency are recognizably rooted in the history of the community in question. With that said, however, the historical and communal determination of a word’s or sign’s meaning does not restrict its use to a predefined community, for the community in question extends to anyone who encounters and learns its use through interactions with other members. The historical trajectory of a community’s interactions, and the conventions, expectations and potential fixities that inhere therein, will be considered further in the course of the paper.

**FIGURE 1:**
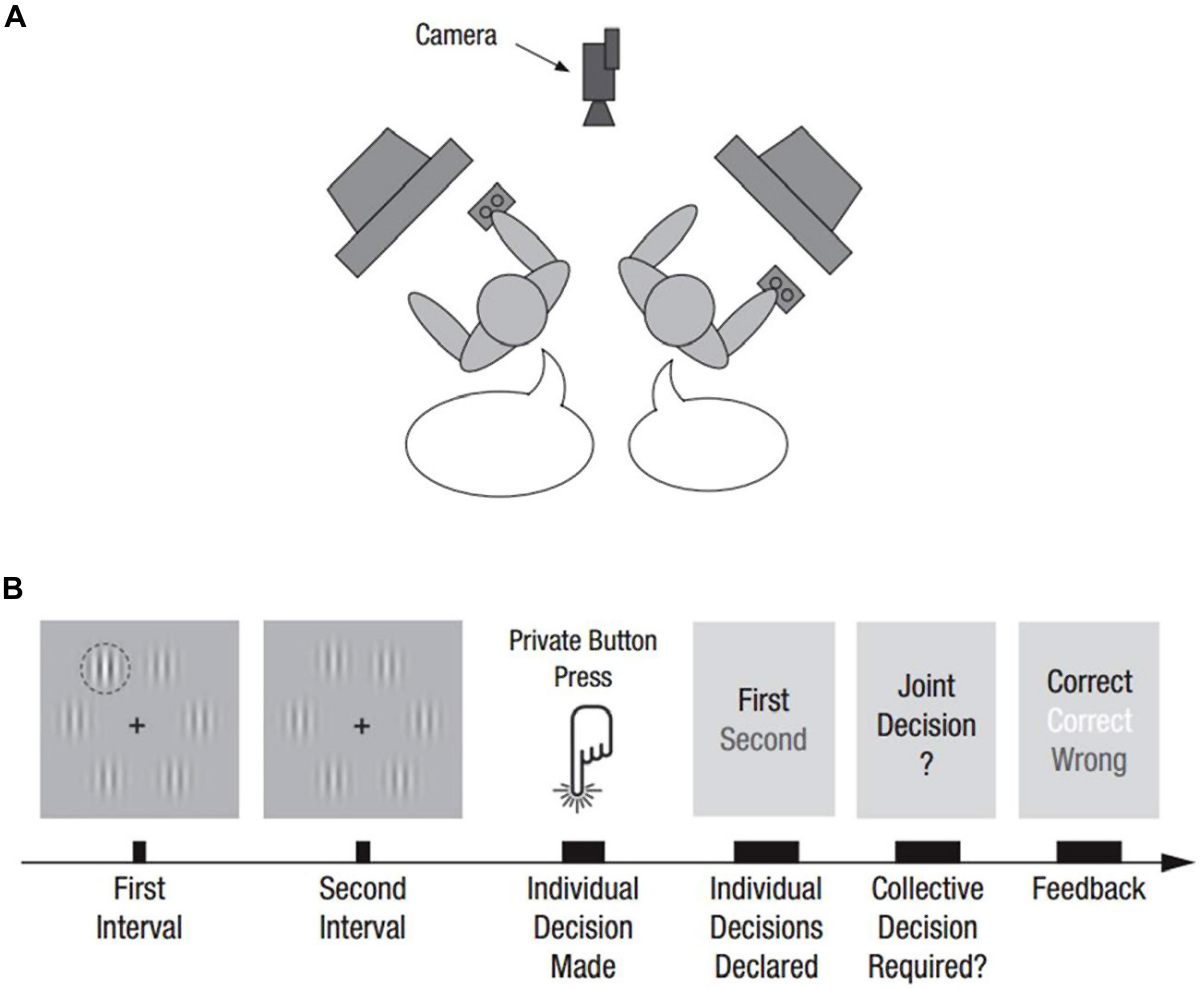
**(A)** Schematic of optimally interacting minds experimental setup. **(B)** Progress of sample trial (from Fusaroli et al., 2012).

In light of this applicability to language more broadly, relevant experiments are not limited to graphical signs and symbols, but also demonstrate the adaptation of natural language under controlled conditions. The aforementioned *Optimally Interacting Minds* experimental paradigm explores the evolution of ordinary verbal language within the constraints of a task situation. Through a series of trials, two people perform a visual discrimination task individually; they do so in the same room, each at their own separate computer. As long as they offer congruent answers (whether right or wrong) they simply precede to the subsequent trial. However, if they give divergent answers, they are prompted to verbally negotiate their joint decision; their linguistic interactions are subsequently analyzed in relation to their performance on the task (see **Figure 1** for a schematic of the *Optimally Interacting Minds* experimental setup). The task thus requires dyad members to, on a trial-by-trial basis, determine who had the more vivid experience of the visual stimulus contrast and submit that person’s decision as their joint answer. Results show that well-performing dyads converged on a common, stable set of terms to communicate confidence, a kind of scale of verbal expressions allowing dyad members to compare their individual levels of confidence. Importantly, general linguistic alignment – that is, the indiscriminate repetition and reinforcement of linguistic forms – failed to positively correlate with performance. Rather, it was the alignment of terms functionally relevant to the task at hand – in this case, conducive to the communication of confidence in discussions of incongruent answers – that was predictive of performance, pointing to the strong context-dependence of linguistic coordination (Fusaroli et al., 2012).

In this process, of converging on a common communicative practice, the seeds may be seen of an implicit *institutionalization* of a particular approach to solving the presented problem. That is, dyads tacitly *instituted* linguistic practices enabling them to better function as a problem-solving system. However, the emergence of such convergence does not in itself constitute an *institution*, in the stricter sense of a social entity in part created and governed by explicit rules. While language in this experiment plays a crucial role in the problem-solving activity, it does not function in the capacity required for the establishment of institutions proper, i.e., by explicit representation or declaration of rules which can be either obeyed or broken. Rather, the use of language here is more akin to actions taken *in* the course of a situation, as opposed to representations *of* a situation.

This distinction may be elaborated by the following contrast. Again, participants in the above experiment communicate their confidence in their answers in a simple perceptual discrimination task; such communication drives their decision-making in direct response to the situation itself, and hence inextricably occurs within the immediate context of that situation. Successful communicative practice – here, convergence on a consistent set of terms to convey confidence – is forged under the selective pressure of the task at hand: well-performing pairs arrive at a means of communicating that *works*, that meets the demands of the situation and affords successful coping within that situation. Yet one might imagine successful pairs informing prospective participants *about* the task they faced, the problems they had to address and solve, and the ways they went about doing so. And perhaps they might proceed to *instruct* future subjects in how to go about responding to this situation, or to situations very much like it. This kind of communication would occur outside of the pressing pressures of the task itself; the task is no longer directly responded to but *represented*, described, to others. With representations of the situation, and representations of how to act in the situation, potential participants would now have something to *conform to*, namely depictions of how to complete the task in a particular way, and something to *comply with*, namely the instructors’ intention that they complete the task in accordance with those depictions. Yet the distinction between *descriptions of* and *prescriptions for* actions must be kept in mind, and while the exact contours of the move from the one to the other may vary from case to case, some general considerations will be sketched and suggested in later sections, including the human propensity toward imitation (Horner and Whiten, 2005).

This transition from transient, emergent coordinative activity to instructions about that activity can serve as an entryway into instituted practices proper. Given situations which select for successful communicative practices, instructions about such situations make explicit what was tacit practice, instructions which can then be followed *correctly* or *incorrectly*. Whereas practices that evolve in response to the selective pressures of a task may *fail* or *succeed* in relation to the task, instructions create conditions in which correct and incorrect actions are possible. The communicative practices of well-performing dyads in the *optimally interacting minds* paradigm can be deemed relatively successful or efficacious, but strictly speaking cannot be considered correct or incorrect, for no standards of correctness yet exist concerning those practices; they are not right or wrong *per se* but more or less functional with regard to solving the problem at hand. Instructions, however, introduce standards of correct practice and action by explicitly representing those practices and actions, and thus give rise to a distinction between conditions of *success* versus *correctness*. However, this stark contrast between implicit practice and explicit instruction, while illustrative here at the outset, belies a more continuous picture involving the gradual conventionalization of communicative practice, in which conditions of correctness come into play prior to, and independently of, the introduction of instruction (e.g., Healey, 2008).

These distinctions, of course, remain coarsely sketched at the moment; indeed the road from tacit habit and practice to explicit institution is a crooked and complicated one (e.g., Fleetwood, 2008), and will be treated more thoroughly in what follows. Moreover, there are certainly cases in which institutional contexts themselves provide the conditions for the emergence of spontaneously responsive practices and actions, and so it would be a mistake to suggest that the trajectory is necessarily unidirectional. However, the focus here is not on how that path happens to proceed in particular cases, but rather on the basic conditions required for the emergence of institutional structures. Suffice it to say at this stage that if one were to take these experimental paradigms as representing some recurring and prevalent set of circumstances, a task or problem situation that people encounter with sufficient regularity and urgency, then it may be fruitfully treated as a kind of microcosm of specialization and, if extrapolated further, institutionalization (Healey, 1997).

Having reviewed some representative examples from this realm of research, we will now proceed to unpack these preliminary observations, and take a closer look at particular implications. In the next section we explicate the notion of *situation* as it applies to these experiments, and examine the role of communication and language therein, a role grounded in the coordination of joint action as opposed to propositional representation. These considerations concerning language will serve to set up what follows, as we address the development of coordination, convention, and, eventually, instruction, in the emergence of institutional interactions, further elucidating the normative distinction between conditions of *success* versus those of *correctness*.

## COMMUNICATION UNDER SELECTIVE PRESSURES: SITUATIONS AND AFFORDANCES

Since the concept of *situation* plays a number of different roles in a variety of domains, we should take stock of the term as it has operated in the discussion thus far. As a start, a situation may be described as a set of circumstances, driven and informed by specific human demands and goals, which in turn exerts pressure on actions performed in accord with those demands and goals. So a situation, in this sense, is at once constituted by human actions and feeds back onto them, is both determined by and determining of those actions. A situation, then, may be provisionally defined as a humanly comprised selecting environment, within which actions may succeed or fail to meet the needs or demands fueling the unfolding of the situation; actions that succeed are selected for, while those that fail are selected out. Of course this is something of an idealization: failed and failing actions often persist despite their repeated failure, for various reasons. For current purposes, however, the idea of a situation as, in principle, determining conditions of success or failure will serve to set the stage for what follows.

In the experiments presented above, communication occurs precisely under such pressing and pressured conditions. In these contexts, the situation is constituted by particular tasks or problems, which participants attempt to address or solve in the course of their activity. Communication here serves to coordinate the joint decision-making of the participants, driving and shaping their actions as the situation unfolds in time. Put more strongly, the communication might be said to *comprise* the situation as a kind of cohering glue, coordinating the participants and partly constituting their joint activity (Demichelis and Weibull, 2008). That is, communication may be conceived as continuous with actions taken within the currently occurring situation, as actions subject to conditions of success or failure, as opposed to propositions characterized by conditions of truth or falsity. While we will not attempt to conclusively argue this point here, in this section we suggest ways in which language may be operating in these settings, as opposed to simply assuming and imposing a reflective, representational conceptualization.

Returning to the *optimally interacting minds* paradigm in particular will help ground some of these thoughts. Recall that participants adjust and attune their confidence by means of linguistic interaction in order to arrive at a shared decision. Here linguistic communication may be understood in terms of the sharing of information, affording access and coupling to the perspective, or experience, of one another (Fusaroli et al., 2014a,b). Thus communication may be viewed as a function of the flow of information through the decision-making system, as an aspect of dynamic informational attunement to the situation. Again, this would be opposed to a view of language as composed of propositional statements analyzable in terms of direction of fit to the world (Price, 2013). Rather, the use of language in this scenario is more aptly conceived in terms of *coping*, employed in direct engagement with a situation, in contrast to a conception of language as somehow standing outside the pressures of a situation, where participants have the space to model or represent the situation independently and to manipulate and control that model.

From this point of view, the status of *situation models*, defined as multidimensional representations of currently unfolding situations (Zwaan and Radvansky, 1998), may be called into question. Such models are often conceived as internal cognitive representations belonging to individuals, and therefore the job of communication is to coordinate and align the distinct situation models of the individuals involved. Indeed, Pickering and Garrod (2004) in their influential account state that linguistic alignment on multiple levels of representation leads to the alignment of situation models. So it seems as if these models are first private and must come to be shared, rather than public and shared from the start. However, Fusaroli et al. (2014a) proceed to question the notion of situation models, understood as internal representations which are aligned by linguistic interaction. Furthermore, communication often is not a matter of simple alignment or matching but rather the achievement of complementary roles and contributions in the course of interaction.

Here we suggest two critical replies to the notion of situation models. On the one hand, language itself may constitute the situation model: rather than merely facilitating the sharing and alignment of internal representations or models, the linguistic interaction, the engagement with the public symbols and artifacts of language, may itself count as the construction and manipulation of a model of the situation (Clark, 1997). On this account, the model, or modeling process, is shared from the start, jointly attended to and co-constructed in the course of communication within the situation. On the other hand, the situation itself, to paraphrase roboticist Rodney Brooks, can simply serve as its own best model (Brooks, 1990). While parts or aspects of the present situation may be modeled or represented, the situation as a whole need not be: the situation is simply *there*, to be attended to and engaged with. From this perspective, linguistic activity serves to guide and direct attention and action in the course of unfolding situations (Richardson and Dale, 2005).

If the situation is directly engaged with during joint activity, without the mediation of situation models or representations guiding that activity, then the situation itself must in some sense be able to direct and constrain that activity. The ecological notion of *affordances* seems a good candidate to account for this, though the term is often subject to loose and various applications. Affordances in the original Gibsonian sense (Gibson, 1977) are functional relations between an organism and the environment it encounters, and hence do not need to be represented and imposed upon the environment. Objects in the environment are perceived in terms of the abilities of an organism to interact with those objects (Greeno, 1994). Thus a pen is perceived by a grown adult in terms of fine motor control by the fingertips; however, an infant who has not yet acquired such fine motor control will not perceive the pen in those terms, but would instead perhaps perceive it as something to grab by the fist and place in its mouth. Affordances then are dependent on the abilities of the perceiver, and those abilities may be in various states of development and transition, with blurry boundaries in between. Therefore the line between what can or cannot be done with an object may be vague and subject to change; affordances are therefore dynamic in relation to a perceiver’s abilities.

Furthermore, while affordances may be understood in this fairly restricted sense of direct bodily engagement with objects – e.g., an object as being graspable in a certain way – the concept is also often applied to possibilities for action more broadly. Here again the line may not be absolutely clear: one perceives a cup as affording *drinking from* because one perceives it as affording *being grasped* in a certain way, a grasp which itself only takes shape in the course of a goal-oriented action such as drinking (Garbarini and Adenzato, 2004). Moreover, there is the question of extending the concept to situations more broadly (Chemero, 2003). That is, given the multiple constraints of a particular situation, can it be said to *afford* certain possibilities for action? If so, then normative considerations will have to enter in, as the range of possible actions within a situation depend not only on the abilities of the actors and the physical features of the objects at hand, but also on a sense of what actions *ought* to be taken given the situation, as well as which courses of action are better than others. The issue remains, however, whether the possibilities for action themselves need to be represented or modeled in some sense, and if so, how such models might be conceived. So it may be said that the notion of affordances affords a range of applications; yet determining when a concept is being extended, or an ambiguity exploited, can be a difficult matter. A more extended exploration of affordances, however, is beyond the scope of this paper; for the moment we may say that the concept offers a possible alternative to the prevalent notion of situation models, and furthermore may motivate a non-representational conception of communication, i.e., a conception of communication as a form of joint action facilitated by the affordances of unfolding situations, rather than necessarily dependent upon or bound up with representations of those situations (Hodges and Fowler, 2010).

As the concept of affordances predominantly applies to interactions with physical objects and artifacts such as tools, it is worth exploring its potential applications to social and symbolic artifacts such as language. Firstly, with a physical tool, an individual in isolation can, in principle, learn and effectively use the tool; the presence of other people is not, logically speaking, required (though of course an individual may encounter physical limitations in attempting to perform a task alone, but that is a separate matter). A single person can rely on and respond to the affordances of the tool – the fact that it is graspable and manipulable in this or that way – and exploit its physical features in interacting with the environment, such as a sharp stick affording throwing while hunting (Heft, 1989). And the standard by which the use of the tool may be deemed successful or not is the intention of the tool user herself, what the user intends to do or accomplish with the tool. A person might intend to use a flat head screwdriver to pry open a jar, and may succeed or fail in the act depending on whether the tool is fit for the task. But while a particular individual may set her own standards in the use of a tool, the same may not be said for the use of language, for the success of communication depends on whether or not one is understood by others (Davidson, 1992). The standards for successful communication are not set individually but communally (Wittgenstein, 1953/2001). So insofar as it makes sense to speak of *affordances* with regard to language, of the possibilities for action that certain words in certain situations *afford*, a social dimension must necessarily be included. The communication occurring in the above experiments, for example, takes place within the context of joint activity coordinated by common goals, so actors may fulfill or fail the intentions of others as well as their own. Yet there is a distinction between *failing with others* and *failing others*; that is, there is a subtle but significant difference between failing jointly with another versus failing another. The latter perhaps implies a power relation of some sort, or at least a distinct stance toward the activity in question, in which the intention of the other must be *complied* with.

In the following section these normative considerations will be elaborated, in terms of the distinction between *conditions of success* and *conditions of correctness*, with uses of language serving as a shifting hinge from one to the other. Our aim in this section, meanwhile, has been to suggest a view of language use under pressured situational constraints, as an alternative to a thoroughly propositional conception. While we don’t pretend to have presented a full account, we have offered possibilities in terms of affordances and joint action, with linguistic communication affording informational coupling and coordination within a dynamically interacting system. A declarative, propositional picture of language may appropriately apply, however, in cases of explicit instruction and compliance therewith. These different uses of language reflect different ways of relating and interacting within and between situations, differences we explore in the remainder of the paper.

## NORMATIVE DISTINCTIONS AND DISCRIMINATIONS: CONVENTION, INSTRUCTION AND INSTITUTION

In previous sections we have introduced a normative dichotomy between *conditions of success* versus *conditions of correctness*. In this section we further specify these distinctions, and complicate the discussion with consideration of processes of convention and conventionalization. We set the stage with a brief recap of the *optimally interacting minds* paradigm, to help ground what follows in a specific concrete case. Recall that pairs that performed better on the visual discrimination task tended to converge on a common vocabulary to communicate relative confidence in their answers: some pairs converged on a confidence scale comprised of visual terms, as in “I think I saw” and “I did not see anything,” while others were voiced in terms of sureness, as in “I’m almost sure” and “I’m absolutely sure” (Fusaroli et al., 2012, p. 4). Ultimately the type of scale used did not matter, as long as they came to tacitly share a consistent practice of communicating their levels of confidence in negotiating a joint response. Thus the demands of the task exerted pressure on participants to communicate in a way that enabled them to fulfill those demands. That is, the experimental setup constituted a selecting environment, comprising a situation defined by conditions of relative success and failure, driving the evolution of actions and practices in accordance with those conditions. Given this situation, participants came to develop a viable vocabulary, specifically honed to cope with the task at hand. Again, here as well as in the other experiments mentioned, feedback and interaction were crucial to the development of these convergent patterns, which arose from the dynamics of the interaction over time, rather than the explicit intentions of the individuals involved (Fusaroli et al., 2014b).

It should be made clear, however, that it is the normative character of the practices themselves that is under question, the normativity *internal* to the practices. This point is important since participants in a paradigm like *optimally interacting minds* receive feedback from the experimental setup as to whether their replies are correct or incorrect. Yet this is a matter of the reinforcement provided by the environment, and hence is *external* to the participants’ practices under those conditions, however much those practices develop in response to that environment. That is, regardless of how the environment feeds back onto and constrains their actions, the actions themselves cannot, under the circumstances, be deemed correct or incorrect, but only more or less successful in adjusting to that feedback and meeting the demands of the task; there are as of yet no standards of correctness in place, and so no way to say that *this* and not *that* particular practice is correct. In other words, while correctness of *outcome* may be said to be in place, in the sense of the right aim or end to be achieved, there is no question of correctness of *practice* as of yet. At this point, the means to be taken remain open, as long as the end is achieved: given a goal, whatever methods or tools that may bring about that goal are acceptable. In this sense the confidence scales arrived at in the *optimally interacting minds* paradigm are tool-like, in that it does not matter which type of scale (e.g., whether in the vocabulary of “vision” or “sureness”) is used, as long as they *work* to meet the same end; thus they demonstrate the detachability of means from ends characteristic of purely tool-like or instrumental relations.

However, the normative status of this coordinative practice, its basic instrumental character in terms of pure conditions of success or efficiency, may quickly become transformed in the course of development. We’ve noted that arriving at a consistent communicative practice is crucial for successful performance. It is then perhaps a short step from this convergence of coordinative practice to the eventual *routinization* and *conventionalization* of such practice. Though a specific procedure may not be explicitly established, procedural routines may emerge and establish themselves in the course of repeated and continual interaction, procedures that may be diverged from or *violated*, and recognized as having been so violated. So it may be that the idea of a starting stage in which there is, strictly speaking, no right or wrong way of going about, where it is purely a matter of whatever works in the context of the task, may be something of an idealization, or at the very least a highly transient phase which undergoes rapid transformation. In other words, though these normative distinctions are conceptually extricable, in the course of actual practice they may well blur together from the very beginning.

A relevant example is found in an experiment by Garrod and Doherty (1994). Here participants jointly navigated a set of mazes either in “isolated pairs” playing together through repeated trials or in “speech communities” where participants would change partner from trial to trial within a closed community. The task was constructed in such a way that participants had to give each other directions and indicate positions in the mazes. They thus had to converge on ways of linguistically referring to positions and routes. Initially participants would generally rely on quite concrete ways of talking about positions in the maze, for instance by reference to the mazes’ figurative properties or by describing the route one would need to go to reach a critical position. However, in the course of the experiment some participant pairs would evolve more abstract coordinate systems (e.g., the chess-like matrix system of specifying a column and row index such as A1 or 3.4) that, once established, proved very effective and transported well between different shapes of mazes. Again, not unlike the reduction of iconicity in previous examples, this development seems to proceed from reliance on the concrete instantiation of the single maze toward a more abstract scheme applicable to all mazes despite their individual shapes and differences. Interestingly, speech communities were more inclined to converge on this more optimal strategy than isolated pairs. Furthermore, and of particular relevance here, in community groups the matrix scheme tended to become conventionalized: the matrix scheme was thus applied even in cases that lend themselves to a more figurative strategy (see also Tylén et al., 2013). That is, even in situations in which a figurative approach would have provided an easier means of reference and direction, the more abstract matrix scheme was nevertheless adhered to. While, in early trials, adaptation to the concrete perceptual stimulus is driving joint linguistic behavior, in later trials the gradual establishment of shared “procedures” comes to override local stimulus affordances: a practice, arising from and rooted in a history of communal interaction, comes to be entrenched and imposed upon the current situation. Still, at this stage, this gradual process of conventionalization proceeds implicitly and only becomes apparent to participants if violated.

This latter point is especially evident in an experiment investigating the development of procedural conventions in a coordination task involving the arrangement of actions and utterances in a certain order (Mills, 2011). Again, participants were organized into small communities, though in this case they communicated by means of a text chat tool. While the referential aspects of the task were made trivial, the experimental situation afforded the evolution of procedures for how and when to share information and coordinate actions. Each participant started with their own list of words, which was not viewable by others. The task was then to submit words from their own list in the formation of one shared alphabetically ordered list. However, they also could not view the submissions of the other. Thus participants had to both communicate their words and to converge on procedures for informing each other which words had already been submitted, and when it was the other’s turn to submit a word. In the course of the experiment, communication within groups grew increasingly arbitrary and rarefied, with progressively abbreviated utterances positioned in highly specified points in the interactions, their meanings, again, determined by the particular histories of those particular communities. These conventional patterns were built up without explicit agreement, emerging from what allows for successful completion of the task, solutions which were then repeatedly taken up, refined, and rendered more efficient.

However, these patterns became explicitly apparent in a critical last trial, where, unbeknownst to the participants, the chat tool would pair up members from half of the communities with partners from different communities: suddenly participants experienced that all the subtle, tacit routines that have evolved with their partners through the course of the preceding trials were violated, bringing them into explicit attention. The manipulation yielded a dramatic drop in performance and participants performed significantly more self-corrections. These observations point to a kind of intermediate stage on the path toward fully instituted practices: despite the highly implicit nature of the interactive procedures, the reactions to violation indicated an emerging normative dimension. For example, one pair of participants may have established a routine in which they would trade turns by indicating the next item for their partner to submit. Meanwhile, another pair may have evolved a tacit routine in which participants would inform their partner which item they had just themselves submitted. When, in that critical last trial, participants relying on such different procedures are unknowingly brought together, their procedures break down revealing their emergent normative character (see **Table 1** for transcript example from Mills, 2011). For instance, in the transcript example below, Participant 2 is expecting to be told which item Participant 3 has just submitted; instead Participant 3 is naming an item that is on Participant 2’s list, thereby following a very different routine. Participant 2’s reaction in line 5 indicates that the breakdown of collective routine is experienced not primarily as *unsuccessful* in meeting task demands, but as *wrong* in a socially normative sense. That is, the exchanges spoke to the violation of norms of interaction, and not merely a struggle with an unfamiliar vocabulary.

**Table 1:** Transcript example from Mills (2011; used with author permission).

Participant3	/APPLE
Participant3	BAR
Participant2	BAR?
Participant3	Yeah of course
Participant2	WHAT?

Cases in which convention overrides local considerations of efficiency and functionality demonstrate the dissociation of conditions of success and functionality from those of correctness. In other words, they indicate the implicit establishment of a certain way of doing things that is not treated merely as an instrumental means to some end. This conventionalization of practice, therefore, opens space for a tacit, emergent sense of *correctness* independent of explicit instruction. It appears that the historical momentum of social interaction to some extent takes precedence over immediate considerations of efficiency and functionality. Though of course in these situations social interaction is itself a significant factor, specifically in terms of the mutual expectations of community members. So while this fixity or conformity of practice may seem in certain specific cases to be inefficient or even detrimental, this conformity may prove functional overall to the extent that sociality itself becomes a major factor in the problem-solving system. That is, there may exist a trade-off between immediate instrumental efficiency and the historical entrenchment of social expectations. Thus the satisfaction of expectations in the course of an activity may override considerations of local affordances.

A revealing ambiguity with the term *expectation* is perhaps worth noting here. On the one hand, it may reflect a neutral attitude toward likelihood or probability, as in *I expect it will rain this afternoon*. This sense is evident in the contrast between *desiring* versus *expecting* something to be the case. On the other hand, it can be used to express an evaluative attitude, as with the *expectations* one may hold for oneself. The question, then, is whether the relevant social expectations are to be understood in terms of adjustments to statistical regularities, to what is likely to happen given particular conditions, or if they are to be understood in the normative sense of what *should* happen, of how others *ought* to act in particular situations. Though these senses of expectation may be conceptually discriminated, they may effectively become blurred in the course of actual interaction. We raise this point not to resolve it here, but to suggest that human dispositions toward social adherence and cohesion may play a role in infusing expectations with normative force (Miceli and Castelfranchi, 2002).

For example, the human proclivity to imitate may be a factor in the establishment and normalization of means and procedures. It is well established that human infants, in contrast to chimpanzees, faithfully imitate the observed actions of another even if some of those actions are manifestly not required for the completion of the demonstrated task (Horner and Whiten, 2005). Whereas chimpanzees disregard irrelevant actions for the sake of efficiency, human children imitate in full despite the cost in efficiency. This tendency to over-imitate, which may have evolved as a channel for the transmission of cultural knowledge (McGuigan et al., 2011), may drive the move from a merely instrumental relation of means to ends to a more conventionalized determination of means and methods. This is one among many cognitive biases that imbue observed behavior with a normative status (Csibra and Gergely, 2009), skewing human development toward conventionality and heightened sensitivity to norms.

In contrast to tacit convention, however, standards of correctness may be explicitly created when others are *instructed* about the task. With the introduction of instructions, actors are expected to comply with the intention of the instructors to have the task accomplished after a certain fashion, in a certain way. The question, then, becomes explicitly one of the right way of going about, of the correct means and method of performing particular tasks. Under these conditions, the actions to be taken are, in significant respects, *represented* by the instructors, representations which serve as the content of imperatives or commands, i.e., *this is how things are done*, *this is how you shall proceed*. Thus the standard to be met is no longer just the successful completion of the task, but of performance according to an explicitly specified protocol. Experimental studies explicitly investigating these aspects of normativization and institutionalization are still quite sparse. However, there are some studies concerning the passing of instructions about procedures among participants. For instance, in recent studies on cumulative cultural transmission, participants acquire a procedure and then have to instruct new participants, who in turn instruct new participants in a “diffusion-chain”-like design (see Mesoudi and Whiten, 2008 for a review). In a representative study, participants had to work together in groups to make paper planes that would fly as long as possible or build the tallest tower of spaghetti (Caldwell and Millen, 2008). Inter-generational exchange was simulated by gradually replacing group members with new ones. Successively, new group members were introduced and invited to contribute to the refinement of current practices. While the focus in these studies so far has been on the accumulation of cultural skill, knowledge, and innovation, such experimental designs can potentially inform discussions on the transition from conventional to fully institutionalized practices.

It is with the introduction of instruction, perhaps, that instituted practices proper come into existence, constituting a kind of endpoint of the continuum we are considering. In this regard the dependence of institutions on the declarative force of language is markedly evident, both in the articulation of representations of actions to be taken and the articulation of the imperative to perform them in that way (Gelati et al., 2002). This, again, is in contrast with the tacit use of language explored in previous sections, where linguistic interaction serves to guide and coordinate joint action in the course of a situation. By comparison, with the *representing* power of language, explicit rules may be formulated that can be either obeyed or broken, correctly or incorrectly followed. Thus tracing the normative transition from implicit practices to explicit instructions is a matter of discriminating the different ways language operates in relation to situations, including how communication both indicates and determines relations of power between people. For instance, as to the source of the power to enforce instructions, as to what enables a person to be in the position to communicate instructions about some course of action, such authority may be derived, at its origins, from knowledge and experience directly (e.g., Kruglanski et al., 2005): the instructors, presumably, know how to go about addressing the problem at hand, having accomplished the task themselves, which justifies their formulation and delivery of instructions. Thus knowledge here is the primary authority, whether practical (knowing how) or propositional (knowing that), which gives instructors the right to speak on the matter, and to not only describe but prescribe actions. An alternative developmental story can be spun of community members describing and discussing ways of going about, arriving at a consensus rather than a hierarchical execution of orders. In this context the role of written language can be seen as especially relevant and stabilizing, investing instructions and declarations with a seemingly permanent, impersonal authority, in contrast to oral commands, conveyed by the impermanent speech of particular persons (Tylén and McGraw, 2014).

## INSTRUMENT AND INSTITUTION

A key theme threading through the discussion above, which we address directly in this section, is the *instituting* of specific means to achieve an end, whereby those means become a *way* of going about. This notion is similar to the Searlian *by-way-of* relation (Searle, 2010): while one may, say, fire a pistol *by means of* pulling the trigger, one votes in an election not merely by means of but *by way of* the ballot box. The pulling of the trigger contributes causally to the pistol’s firing; by contrast, submitting a ballot itself *counts as* the very act of voting, and is not to be understood as an instrument toward some separate end. But whereas for Searle such institutional facts necessarily depend on collective beliefs, we treat the selection and institution of practices in terms of their gradual and tacit establishment, a process driven by human disposition and action as opposed to propositional attitudes.

Central to this process, we claim, is the emergence of an implicit sense of *correctness* above and beyond instrumental *success*, which, as discussed earlier, may arise through *conventionalization*. With convention, a certain pattern of practice is established, by virtue of which deviation is possible; establishing a pattern enables the possibility of breaking it. Though again, in the case of human interaction, such a pattern isn’t a matter of simple statistical regularity, of the assessment of, and adjustment to, probability: rather there is a normative character to the persistence of the pattern and the expectation inhering therein (Miceli and Castelfranchi, 2002). This is particularly the case with communicative practices, given the necessary involvement of, and negotiation with, others. And as we hope has been demonstrated by our review above, conventions need neither be explicitly established nor explicitly acknowledged: neither the origin of nor the adherence to convention requires explicit deliberation. Rather responsiveness to convention may be seen as akin to a kind of perception, as a sensitivity to temporally extended patterns of interaction, a sensitivity cultivated by participation in those patterns. Through this recognition of patterns comprised of communal histories of interaction, a tacit sense of correctness is instituted, a sense of a more or less right *way* of doing something, relative to the community one engages in.

Establishing conditions of correctness has a number of significant implications. Firstly, when a particular means of achieving an end has been established as a *way* of accomplishment, as a *style* of doing characteristic of a community, such instituted activity can become an “object,” so to speak, of joint attention, a temporal structure around which to coordinate. Conventions, as reliable patterns of interaction, can serve as coordinative structures in the course of an activity, facilitating its flow (Alterman and Garland, 2000). One effect, then, of conventionalized practice is to simply make interactions more streamlined and efficient in this manner.

Secondly, and perhaps more profoundly, conditions in which it makes sense to say that this or that act is *correct*, beyond how successful it might be, enables the conveyance and detection of significance in a way that mere instrumentality wouldn’t allow. For, if conditions and considerations of success, solely and strictly speaking, were all that were in play, then, in principle, any change of procedure, any alternate act taken in the course of some goal, would be treated instrumentally as merely another means toward that goal. Whether these changes would prove fit for purpose is another question: some may be tossed aside as inefficient or unfeasible, which speaks to their instrumental dispensability. Since no grip exists in the means themselves, they would be in principle interchangeable, assessable only in terms of their instrumental success, their status as mere means to an end. There would be no sense of different means *meaning* different things, as they would all be defined by the goal-driven constraints of the given situation.

However, if correctness of practice is established, if the means are in some sense fixed, and become a *way*, then variation may be treated as violation, divergence deemed deviation. Under such conditions, difference in action may take on special significance, beyond being another means to be dispensed with or disposed of. This, again, is bound up with the transformation from *instrumental* to *instituted* practice. For, with instituted practices, conditions in addition to those of success are introduced, enabling a further sensitivity to differential activity. The question becomes one not only of outcome or goal, but of the character of the practice itself. And when actions are no longer only means in relation to an end, no longer determined solely by their aim, variation can become meaningful within and against such instituted patterns of interaction. This is not to say that change of practice, under these conditions, isn’t possible, but that such change would be treated, at least initially, as a violation of current norms of interaction. Change would thus undergo some normative process of negotiation, whether explicit or implicit, and not just practical adjustment, which speaks to an important dynamic between fixity and flexibility of practice. This is also not to say that conditions of success and correctness are somehow opposed or separate: they are very much interrelated, and our concern here has been their conceptual disentanglement, however much they may be a tangle in fact.

These themes are especially pertinent to linguistic interaction. Many have noted the necessity of conditions of correctness for linguistic meaning. Consider, for instance, the centrality of normativity in the work of Wilfred Sellars (e.g., Sellars, 1956/1997), for whom the establishment of norms of correctness is crucial to our capacity for conceptual thought and our operation in the “space of reasons.” Consider as well the work of Donald Davidson, for whom conditions of correctness and truth are central to the very possibility of thought and content (Davidson, 2005). In the essay “Truth Rehabilitated,” Davidson speculates on an infant’s growing entry into language. In the early learning stages, the child, he says, “is still a pragmatist” (Davidson, 2005, p. 15), concerned with the consequences of its vocal behavior, whether in the form of reinforcement from others, or in the attempt to attain something through others. From the perspective of the teacher, already a master of the language, the child is being taught the meanings of words and phrases; from the point of view of the child, linguistic engagement is purely a matter of result and outcome. It is with the dawning awareness of the possibility of being mistaken, that this or that word may be applied correctly or incorrectly, that the child starts to have a sense of the meanings of the words being used. For this possibility for error is not merely a matter of failure: a word is wrong not because it somehow fails to work on some occasion. Rather a word is right or wrong because its use has been established or *instituted* as such. There is much more to be said on this subject, of course. Suffice it to say that, with the introduction and *institution* of correctness, the instrument of language is no longer merely instrumental but intrinsically meaningful, in its sensitivity to correctness and the violation thereof.

Here we should acknowledge the use of natural language in many of the experiments reviewed, and hence the prior presence of conditions of correctness. Thus the normative transition described above, from conditions of success to correctness, occurs within a frame in which a basic sense of correctness is already in place. However, a distinction may be made between the material of language itself, conditioned by conditions of correctness, and the particular linguistic practices that develop from that material. The latter may be more or less successful depending on the situation, and may themselves come to be instituted as correct communicative practice. In this light the emergence of communicative practices may be viewed as recapitulating the transition from conditions of success to those of correctness characteristic of the institution of language itself.

## CONCLUSION

In this paper we’ve traced the emergence of coordinative, conventional, and institutional interactions in terms of the transformation of our *normative* engagements, a process inextricably involving variations in linguistic and communicative practice. This instituting of communicative practice provides a conspicuous opportunity to investigate the variety and interdependence of our normative relations. A normative context must exist for this process to get a grip, a setting in which success or failure is possible, selecting for certain words and communicative forms that *work*, which are functionally suited to a problem situation. The tools of ordinary language are brought to bear to address a problem, and refined and retooled in the process, forging a vocabulary transitioning from the everyday to the specialized, from the common to the honed for the task at hand. And with the emergence of convention and the introduction of instruction comes an instituted environment that not only selects but *sanctions* certain actions, constituting a significant normative shift in social organization.

In charting this course from conditions of *success* to those of *correctness*, we started with the stark contrast between implicit coordination and explicit instruction, in order to clearly introduce and elucidate the normative distinction. We then explored the continuum between these two poles, in the form of the emergence of convention and the establishment of tacit standards of correctness. We also touched on potential dissociations between the two, both in the sense of conditions of success existing prior to and independently of correctness, as well as the possibility of conditions of correctness coming apart from those of success. The latter is evident, and perhaps familiar, in the case of practices and procedures, deemed or instituted as correct, no longer working efficiently; that is, though officially considered correct, they may well have become dysfunctional and unsuccessful.

This normative perspective provides a way of characterizing processes of institutionalization. From this stance, practices become *instituted* when they are established as correct above and beyond their instrumental success. So while certain practices are *selected* under conditions of success, they become *instituted* under conditions of correctness, whereby mere *means* become *ways* of doing. And as a terminological aside, perhaps the verb form *institute* (as in *instituted* practices or *instituting* activities) is more aptly applied to cases of implicit correctness, in which the processes retain a degree of fluidity and informality, whereas the nominalized *institution* may be best reserved for social structures constituted by the articulation and declaration of formal and explicit rules.

Again, we’ve been keen to proffer a conception of linguistic interaction as basically coordinative rather than representational. Such a view points to a role for language in the instituting of interaction that does not depend on the idea of declaring institutional facts into existence, of creating institutional reality by performatively representing it as such. Rather communicative and linguistic practices enable and facilitate coordinative activities (Maturana, 1978). Furthermore, being essentially social, linguistic practices may be especially prone to normativization, and hence serve to consolidate coordination. Indeed these normative transformations are themselves inherent to language: language by its nature is a dynamically *instituted* and *instituting* phenomenon.

The experimental semiotic frame here surveyed is especially applicable to these processes, as it treats signs and words as living forms within local environments, adapting to the selective pressures of specific scenarios and problems, with success and failure a matter, as it were, of life and death. Hence the emblematic nature of something like the *optimally interacting minds* experiment, which serves as a microcosm of the selective environments that foster adaptive communicative activity. Indeed there appears to be a kind of double adaptation at work: not only do communicative and linguistic actions adapt to the task environment, but people come to adapt to the developing linguistic environment as well, by aligning with and adopting the communicative forms employed. Thus a vocabulary develops to adapt to a problem, creating a linguistic environment which in turn is adapted to. In this light institutions can be seen as informed and controlled communicative environments designed for the consideration and solution of specific societal problems.

Crucially, the aims and ends of an institution are themselves articulated in terms of the language of the institution itself. The language of an institution to a certain extent constitutes the possibility of its aims and goals. For example, the possibility of convicting someone of a crime is constituted by legal institutions: the legal system, in its institutional articulation, is not merely an instrumental means of achieving the goal of finding someone guilty, but rather constitutes the very possibility of that goal. Language in this sense may be viewed as a kind of cultural technology, enabling the opening up of conceptual possibilities (Clark, 1996). And while focusing on the tool-like aspects of language may offer insights (e.g., Tylén et al., 2010), emphasizing the efficiency and instrumentality of linguistic interaction, a focus on the instituting and institutional aspects of language needs to enter in as well; for there is a difference between making things easier and making things possible to begin with.

Finally, the experimental work reviewed exemplifies the ways in which the broader resources of natural language are brought to bear on certain situations. Indeed, we always find ourselves situated in specific situations (Gallagher, 2012), which are always informed to some degree by direction, purpose or functionality, whether in the form of an explicit aim or goal, or more implicitly and indefinitely. Our ordinary language, in its varied and variegated vocabulary, has evolved, and continues to evolve, in response to fluid, multifarious circumstances. Just as these experiments illustrate the shaping of communicative activity under the selective pressures of contrived and controlled experimental conditions, so too has natural language been forged under pressures to cope with a vast and various range of situations, selected under shifting conditions of success and failure, with the survival of the fittest forms for those situations. To articulate the evolutionary perspective explicitly: words that work *live*, continuing in circulation and continually reproduced, while those that do not work, that fail to serve, *die*, falling out of use, and no longer reproduced. And while language adapts to human environments, to situations constituted by human needs, we, of course, adapt to our environment by way of language, in turn further informing our environments in the creation and differentiation of our diverse social milieux.

## Conflict of Interest Statement

The authors declare that the research was conducted in the absence of any commercial or financial relationships that could be construed as a potential conflict of interest.
